# Exploring the Qualitative Experiences of Administering and Participating in Remote Research via Telephone Using the Montreal Cognitive Assessment-Blind: Cross-Sectional Study of Older Adults

**DOI:** 10.2196/58537

**Published:** 2024-11-15

**Authors:** Shirley Dumassais, Karl Singh Grewal, Gabrielle Aubin, Megan O'Connell, Natalie A Phillips, Walter Wittich

**Affiliations:** 1 École d'Optométrie Université de Montréal Montréal, QC Canada; 2 Department of Psychology University of Saskatchewan Saskatoon, SK Canada; 3 Department of Psychology Concordia University Montreal, QC Canada

**Keywords:** neuropsychological tests, telemedicine, social inclusion, telehealth, remote, qualitative, neuropsychological, cognitive, screening, assessment, perception, perspective, telephone, cross-sectional, thematic, mobile phone, Montreal Cognitive Assessment, MoCA

## Abstract

**Background:**

The COVID-19 pandemic caused a drastic shift in the practice of research and clinical services. It has been noted that cognition measured via in-person versus remote methods differ substantially, and it is possible that subjective and experiential differences exist between modalities.

**Objective:**

The aim of the study is to explore the perceptions of both researchers and older adult participants on the experience of remotely conducted research using a cognitive screener.

**Methods:**

We conducted a thematic analysis of the experience of engaging in remote research from both the participant (n=10) and researcher (n=4) perspectives. The research interaction was framed through teleadministration of the Montreal Cognitive Assessment-Blind (suitable for telephone administration) and administration of a subsequent semistructured debriefing interview. Participant perspectives were garnered during debriefing interviews, while researcher insights were collected via self-reported qualitative field notes completed following each research session.

**Results:**

Data aggregated into themes of barriers and facilitators from the lenses of both participants and researchers. Participants noted facilitators including short instrument length, convenience, and presession contact; barriers included the length of the interaction, some tasks being more challenging on the phone, and the potential for participant dishonesty. Research assistants noted several facilitators: instrument length, rapport building, ability to prepare for and record sessions, and comfort with the protocol; barriers were items with too many response options, telephone issues (eg, response delays), and concerns about participant comprehension.

**Conclusions:**

These results suggest remote telephone-delivered cognitive screening tools as a feasible and acceptable method of research inquiry. The findings provide a starting point for the inclusion of diverse populations in research to capture underrepresented groups whose input would immensely benefit our understanding of remotely delivered cognitive screening measures. Further, we offer materials (eg, checklists), which can be used in future investigations to promote future inclusive research and increase generalizability.

## Introduction

Though research on remote cognitive assessment extends back many years [1], the COVID-19 pandemic accelerated research in this domain [2]. There is little doubt that this remote research has provided immense benefit, and it is critical to understand the perceptions of people on both sides of these interactions to inform the development of best practices for research, which may then inform clinical practice in the future. The Montreal Cognitive Assessment (MoCA) is a commonly used screening tool for cognition, and remote administration methods have been investigated previously [3]. However, little attention has been paid to the qualitative aspect during administration. In this study, we explored the perceptions of both researchers and participants on the experience of remotely conducted research using a cognitive screening tool. As such, we administered the MoCA [4] adapted for people with vision impairment (MoCA-Blind [5]) via the telephone. We chose the MoCA-Blind, given evidence that the telephone is a widely available device among older adults [6]. To supplement the capturing of this practice, we explored experiences and preferences related to general telephone use through a closed-ended questionnaire.

Research into remote cognitive assessment research has roots in the 1990s [1,7], and studies continue to examine the utility of this model [8-10]. Benefits of remote methods have been noted to include increased convenience and access [11], improved safety (eg, limited COVID-19 exposure), and management of costs [12]. Remote methods have been deemed feasible and acceptable by both providers and patients [12-14]. However, concerns about remote methods have been described. For example, a digital divide (ie, availability of technology, internet connections, or digital knowledge [15]) can prevent access [16] and has been associated with additional psychological barriers for people attempting to engage with technologies, such as apprehension or anxiety [17]. Despite these trepidations, a recent investigation provided preliminary evidence toward a potentially useful telephone-delivered 10-item cognitive screening test [18]. Further, recommendations have been made to facilitate appropriate remote assessment [19] such as ensuring the correct infrastructure (eg, videoconferencing or telephone), and that appropriate tests and adaptations are considered.

In-person and remote assessment of cognition differ substantially, each associated with different assumptions [10,20]. For example, in-person norms may not be appropriate for application to remote assessment [21] because standards developed for one modality may not hold for the other [20,22,23]. Acknowledging this difference between the 2 modalities underscores the need for further evaluation of remote methods; if there are objective concerns, are there subjective and experiential ones as well? For example, turning to remote methods could introduce uncertainty to the administration, change the testing environment (eg, lack of visual cues such as smiling or nodding), or require an increased effort on the part of the participant [24,25]. These issues have implications for the cognitive data researchers generate and their subsequent conclusions.

Pertaining to the MoCA, there have been several recent investigations into its remote administration. For example, a recent study compared face-to-face and videoconferencing administration of the MoCA following stroke, reporting preliminary evidence of equivalency [26]. In addition, the telephone MoCA was found to differentiate mild cognitive impairment from cognitively normal older adults in a diverse, community-residing cohort [3]. Lindauer et al [27] reported excellent reliability of the MoCA when used with direct-to-home telemedicine, with participants and clinicians reporting this as a feasible option for assessing cognitive functioning. Additionally, remote administration of the MoCA via videoconferencing has been received positively by participants with movement disorders for reasons of reduced care partner burden and lessened commute [28]. It is worth noting that these studies probed questions of reliability, equivalency, and to some degree feasibility through primarily quantitative means (eg, statistics, completion rates, and Bland-Altman plots). There has been little explicit attention given to more detailed qualitative analyses.

As demonstrated earlier, the MoCA is a commonly used research tool for assessing cognition remotely. As this research evolves, so too have considerations pertaining to remote delivery. Several challenges have been recently identified with a shift to remote administration of the MoCA [25]. First, the interpretation of data from remote administration requires a comprehensive understanding of the examinee’s vision and hearing abilities. In addition, test administration would lack standardization due to the heterogeneity of devices used to deliver (examiner) and receive (examinee) the information. The lack of standardization may also occur at the level of altered stimulus delivery or misperceptions of instructions or examinee responses. Taken together, delivering and interpreting a remotely administered MoCA test score are replete with challenges and pitfalls and must be used cautiously [24]. Due to these challenges, it has been suggested that a remotely delivered MoCA be used as a familiar vehicle to elicit qualitative behavioral observations, generate and test hypotheses, as well as plan care delivery [25]. Considering these discussions, it is important to understand the experiences of giving and receiving the MoCA remotely to form a base on which to build research judgments and interpret our data accurately.

A recent study explored experiences of remotely delivering the telephone-administered MoCA-Blind as an eligibility tool for a randomized control trial [29]. Researchers reported uncertainty, being unsure whether cognition or hearing was at the root of difficulties. Pragmatic concerns also emerged, such as issues with participants physically holding the phone for an extended period or distinguishing a requested tapping response from background noise. Finally, there was uncertainty about the impact of the testing procedure, as the researchers were only privy to what participants shared over the phone (eg, no visual warning signs of fatigue or mood). Thus, it seems clear that there are experiential differences in the MoCA-Blind when given remotely that deserve more attention for the benefit of researchers, clinicians, and participants.

To better understand the experiences of older adults and researchers who participate in research involving cognitive screening over the phone, we administered the MoCA-Blind in a structured research interaction. Our focus on older adults stemmed from them being a substantial group of individuals for whom the administration of the MoCA is increasingly relevant [10]. Given the differences that have been articulated in the literature regarding in-person and remote methods, it stands to reason that the experience of researchers and participants may also differ qualitatively. To explore and better understand this experiential phenomenon, we examined the dyad of test administrator and participant in the context of remote cognitive screening and questionnaire-based interactions. The research assistant conducted a phone interview with the participants regarding their research participation experiences, including feasibility information such as barriers and facilitators [30]. Research assistants also documented their perspectives through field notes. These data sources were explored through separate thematic analyses to elucidate areas of common ground and uniqueness. This paper presents the methodologies and findings from 2 studies conducted to investigate the perspectives of participants (study 1) and researchers (study 2).

## Methods

### Ethical Considerations

Ethics approval for research involving human participants was obtained from the institutional review boards of the Université de Montréal (CERC 2021-394), the Centre de Recherche Interdisciplinaire en Réadaptation du Montréal Métropolitain (CRIR-1493-0720), the Centre Intégré Universitaire de Santé et de Services Sociaux du Centre-Ouest-de-l’Île-de-Montréal (MEO-50-2021-2583), and the Centre Intégré de Santé et de Services Sociaux de la Montérégie-Centre (MP-50-2022-1262). Verbal informed consent for study participation was obtained from participants over the phone and recorded. No compensation was offered to participants. The data presented in this paper have been deidentified.

### Study 1: The Experience of Remote Research From the Perspectives of Participants

#### Participants

Participants (n=10) were recruited from a bank of individuals at the Institut Universitaire de Gériatrie de Montréal who volunteered to be contacted to participate in research studies. Inclusion criteria consisted of a minimum age of 65 years, no history of cognitive impairment, and the ability to read and communicate in either French or English. After the Institut Universitaire de Gériatrie de Montréal provided our research team with the participant bank, individuals were contacted via phone. Those who were available and interested were then recruited as a convenience sample.

#### Materials

A telephone-use questionnaire was administered, which was developed by our research team to assess participant comfort with the phone modality as well as each participant’s technological circumstances (eg, device used; Multimedia Appendix 1). In light of the social and health context during the period of our study, we also administered a COVID-19 questionnaire, which is available in Multimedia Appendix 2. Subsequently, the MoCA-Blind was administered remotely, following published adaptations and guidance [5]. The MoCA-Blind omits the subtest items that require a functional vision for its completion, such as the clock-drawing task. Further, the tapping portion of the attention subtest was adapted, whereby participants had the option of tapping on their phones with either their hand or a pen. Testing sessions concluded with a semistructured debriefing interview (Multimedia Appendix 3), where participants shared their experiences during remote research. This interview guide was developed by the research team using a phenomenological approach [31].

#### Procedure

This study was conducted in the summer of 2021 in the context of pandemic-related lockdown regulations in Quebec, Canada. Following recruitment, initial phone contact consisted of obtaining verbal consent and the administration of the telephone-use questionnaire. Subsequently, each participant received a checklist of questions and reminders by email to help troubleshoot any problems, such as their use of a hearing aid, how they planned to use the phone, and ensuring they had the necessary technical tools and a suitable environment for the protocol tasks (eg, no background noise and being alone). A second phone call was made within the subsequent 2 weeks, where checklist items were revisited with participants to ensure a smooth administration of the protocol. Following this, the MoCA-Blind and the semistructured debriefing interview were conducted over the phone. During the intervals between different segments of the phone call, verification questions were posed to ascertain the audibility and modality of telephone use. Phone calls were recorded using NoNotes (NoNotes Inc), a third-party phone call recording app. Recorded testing sessions, note-taking, and subsequent transcription facilitated scoring and qualitative coding of textual data. All interviews were transcribed verbatim and analyzed by 2 research assistants on Microsoft Word. Given the bilingual context of research in Quebec, Canada, some interviews were translated from Canadian French to English using the DeepL software (DeepL SE). The resulting translations were proofread by a fluently bilingual member of the team to maximize the preservation of the meaning of the original interviews. All participant information (eg, audio recordings, contact information, and test results) were identified by an ID number to preserve confidentiality.

### Study 2: The Experience of Remote Research From the Perspectives of Researchers

#### Participants

Research assistants (n=4) were recruited from a laboratory at the Université de Montréal’s School of Optometry focused on sensory-cognitive aging. Given Quebec’s bilingual population, research assistants are required to be functionally fluent in French and English to conduct testing sessions in accordance with participants’ language preferences.

#### Materials

A Microsoft Excel sheet was created for each research assistant to report qualitative field notes after each completed testing session. This form was divided into 2 sections. The first section pertained to the observations made during the testing sessions, such as the length of the call, participant behavior, problems encountered, and troubleshooting solutions. The second part centered on personal experiences when testing participants over the phone, such as how comfortable research assistants were testing over the phone, their opinion of the phone modality, and test session facilitators. Additionally, research assistants individually reported information on their environmental setup during remote administration, including information about how they used the phone (eg, phone to ear, on speaker, and on headphones; Multimedia Appendix 4).

#### Procedure

The research assistants provided written informed consent and completed a demographic questionnaire (Multimedia Appendix 5). After each completed test session, they reflected on the operational aspects of the study using the previously described Microsoft Excel sheet to detail qualitative insights. All testing sessions were conducted in French, over the phone, and were recorded using the NoNotes software.

### Analyses for Studies 1 and 2

A multistep thematic analysis [32] was conducted on the 10 participant interviews, and the 10 sets of field notes generated by 4 research assistants. The analyses were guided by the highly cited framework of Bowen et al [30] for feasibility. This publication does not provide a definition of feasibility per se rather delineates 8 areas of focus for feasibility studies including acceptability, demand, implementation, practicality, adaptation, integration, expansion, and limited efficacy testing. The authors further define implementation as the extent to which a service can be delivered as planned, which is synonymous with fidelity. As such, our coded responses related to ways in which the research experience could be evaluated and organized [30]. These included areas relevant to feasibility such as acceptability, demand, practicality, implementation, and efficacy [30]. Our descriptive method was low inference, intended to generate a summary of events in everyday terms [33].

Each data source (ie, interviews and reports) was analyzed by 2 independent research assistants. To begin the analysis, 2 of the research assistants who conducted the testing sessions read and reread qualitative data to facilitate data familiarization. Importantly, during this initial phase, each research assistant examined interviews they did not conduct themselves. Subsequently, interviews and reports were examined again, generating the initial codes. They then exchanged data files such that each interview and report were analyzed twice. Throughout coding, both these research assistants engaged in reflexive memoing to provide an audit trail for code creation [32]. The resultant codebook was further refined during iterative collective exchanges to address any discrepancies. Finally, codes were reviewed with the larger interdisciplinary team to identify and define emergent themes through consensus-based iterative discussion.

Several methods to improve rigor were used in each of the thematic analyses [34]. First, the interviews were conducted using an iterative approach, whereby each research assistant posed follow-up questions on comments made by the participants to increase the in-depth understanding of their meaning. To support confirmability, a comprehensive audit trail was kept to document notes about the context of the research, methodological decisions (eg, code book revisions), and the thematic analysis process [34]. The research assistants who conducted the testing sessions noted any salient and interesting information they observed and any methodological decisions they made in the process. This note-taking initiative also facilitated credibility, as it ensured attention to reflexivity and the acknowledgment of potential biases [35,36]. Furthermore, ensuring that the researchers did not perform the preliminary analyses of their own data decreased subjective bias. In addition, our supervising research team was diverse, including expertise in aging (NAP and WW), neuropsychology (NAP and MO), sensory impairment (WW), and qualitative research (WW and MO), lending depth to the study’s development, data collection, and analyses. Finally, we used low-inference methods during analysis to authentically convey participants’ experiences, adding further confirmability [34]. The COREQ (Consolidated Criteria for Reporting Qualitative Research) checklist for this study is available in Multimedia Appendix 6.

## Results

### Study 1: The Experience of Remote Research From the Perspectives of Participants

#### Participant Characteristics

The sample consisted of 8 women and 2 men, all older adults (mean_age_ 69.33, SD 12.20; range 67-76 years); information on participant gender was provided by the recruitment agent at the Institut de Gériatrie de Montréal. Information on participant education level was not collected. Results of the telephone use questionnaire indicated that half of our participants (5/10) felt technology to be very important, while others reported that it was important (3/10) or of little importance (2/10). Participants reported making phone calls almost every day (4/10), every day (3/10), or once a week (3/10). None of the participants reported requiring assistance when making these phone calls, but 2 reported encountering technological issues when using their devices. In total, 5 participants reported primary use of their mobile phone, 4 reported using their landline phone more often, while 1 used both equally. A total of 9 participants obtained scores equal to or above the adjusted cutoff on the MoCA-Blind, while 1 participant obtained a score of 16, which was outside the normal range using the adjusted threshold of 18/22 [5].

#### Thematic Analysis: Participant Perspectives

#### Facilitators for Remote Methods

Broadly, participants viewed the process of remote research as acceptable, thereby suggesting telephone administration to be appropriate for use in research. When discussing the MoCA-Blind, participants considered the instrument suitable for remote administration because of its short straightforward test instructions and items*.*

It works out well. Also, maybe because I knew the tests you were going to have me do.

Participant 1

Participants reported a demand for telephone-based research methods, citing its convenience as facilitative to participation:

If there is a snowstorm, and the situation on the roads is difficult, even if I travel by subway, I will prefer to do it at home.

Participant 2

In addition, several participants described the appeal of participating in research from the comfort of a personal environment (practicality), as it allowed for better time management, was convenient, and minimized distractions stemming from the laboratory environment (eg, formality of environment). Conducting the study’s tests in a familiar environment encouraged feelings of relaxation and physical comfort and increased concentration and stimulation:

It’s like I was saying, given the nature of the environment and the fact that there are no distractions around me, well, that creates a context that is favorable to concentration, to paying attention and to exercising the cognitive function that is associated with it.

Participant 5

Participants acknowledged several aspects of our research method as facilitative to implementation. First, they reported feeling efficiently prepared for the testing sessions because of the reminder phone calls and checklists (eg, questions pertaining to hearing aid use and adherence) used by our team. This preparedness allowed participants to plan their schedule around the study participation, troubleshoot technology, arrange their environmental setting, and prepare for the session both mentally and physically. Additionally, they acknowledged and valued our research team’s punctuality, respect, trustworthiness, professionalism, and organization.

I think that your way of doing things is also important in a telephone conversation because even if we don’t know each other, it’s as if I feel a relationship with you.

Participant 2

Well, I had taken notes when you asked me these things. Then, I reread that just a few minutes before the call today then I realized that my battery was low.

Participant 3

#### Barriers to Remote Methods

Although participants deemed the telephone modality as appropriate to conduct research, there were several potential barriers to its implementation. First, there was concern about the length of remote research activities. Participants emphasized limiting the length of the research session, describing the consent process as one area that could be shortened. Despite the efforts of the research team to expedite the process, the emphasis placed on obtaining consent over the phone was identified as being long and tedious:

I think you put a lot of emphasis on consent. I don’t need so much emphasis because once I’ve read it (prior, by email) and you’ve summarized it in two or three sentences, it’s fine. Maybe for someone who is a first-time participant it’s more important, but I find that a bit long.

Participant 3

Furthermore, some portions of the MoCA-Blind were identified as challenging in a remote context. Concern was raised for the attentional tapping task, as the phone presented some distraction and apprehension.

I had a little concern at one point during the test because when I was tapping for the A’s on my phone. Well, my phone was in my ear, but I could see that there was some activity there, visually, on my phone. I was afraid that it would cut off there.

Participant 3

One participant articulated a preference for in-laboratory research, citing a perceived absence of stimulation in her home setting. Similarly, other participants noted an absence of personal connection as well as confidentiality as issues with the telephone approach. The telephone also appeared to create some apprehension, as participants feared the occurrence of technological issues such as a bad phone connection or lack of audibility. Interestingly, one participant suggested the use of videoconference platforms as an alternative to telephone-based methods, with the aim of enhancing interpersonal interactions during the research process.

I concentrate better when I see the person in front of me. I’m less distracted actually. I concentrate better. It’s more stimulating.

Participant 4

Well, it is sure that the contact is less interesting by phone. It takes something away from the human contact. It’s more impersonal. Then I could end up losing interest (in remote research).

Participant 6

When the questions are very personal, you have to know who you are talking to and what the person will do with the information.

Participant 1

If it was by Zoom, it would already be better, I would be a little less suspicious, because I would have associated a face with a name.

Participant 1

Well, it distracts me and at the same time it annoys me a little because I can’t hear as well on the phone as I can in person. That is to say, my phone doesn’t have an extraordinary sound ... Maybe we could have the image at the same time. Or my phone should have better sound.

Participant 4

Finally, 2 participants indicated the potential for inaccurate data due to cheating due to a lack of supervision and the accessibility of materials such as writing tools and paper.

And then I pulled out a piece of paper in case I had to use it. I was tempted to use it there when you asked me to memorize but I didn’t.

Participant 3

On the phone, we could cheat the memory test. If someone writes it on a paper, it is easy to repeat.

Participant 1

### Study 2: The Experience of Remote Research From the Perspectives of Researchers

#### Participant Characteristics

Four research assistants were recruited from a research laboratory from the Université de Montréal’s School of Optometry (mean_age_ 29.75, SD 12.20; range 23-48 years), comprised of 3 women (2 master graduates, including author SD, and 1 undergraduate) and 1 man (laboratory coordinator). All research assistants self-reported their gender. French was the first language for 3 of the research assistants, while Portuguese was the first language of the fourth; however, all were functionally fluent in both English and French.

#### Thematic Analysis: Researcher’s Perspectives

#### Facilitators for Remote Methods

Research assistants noted several aspects of the research process as important for facilitating successful implementation. First, they denoted the importance of administering tests and questionnaires with minimal response choices as well as clear and simple instructions to promote *acceptability*.

The instructions are very “phone friendly” and did not cause any confusion for the participant.

Research assistant 3

In addition, all research assistants noted that sessions were affected by the rapport between the research assistant and the participant. Specifically, sessions were perceived as more fluid when the participant appeared to be engaged and interested in the research. Further, one research assistant related participants’ skillfulness with modern technology (eg, smartphones) also facilitated the process through flexibility and convenience.

The participant was already accustomed to telephone research and uses an iPhone which I assume helped the course of this remote study.

Research assistant 3

Recording the research session was also described as a desirable aspect of remotely conducted research. One research assistant expressed their intention to review the recorded sessions to facilitate increased proficiency in future testing sessions.

I will listen to the recording to identify where I need to improve next time.

Research assistant 4

Research assistants further explained that remote research methods were practical and implementable, provided that appropriate preparatory measures are taken. For example, presession organization was identified as a key facilitator, including preparing necessary documents as well as charging and troubleshooting technology (eg, telephone and recording software) to prevent any potential issues.

I wrote down and organized all the instructions and possible dialogues for the testing session in a file. This helped me tremendously in conducting the tests.

Research assistant 4

Relatedly, research assistants were able to arrange their own environmental settings according to their preferences. This allowed them to frequently create a calm and distraction-free environment. Importantly, the use of hands-free technology was considered essential to enhance physical comfort and ease of manipulating materials during the research sessions (eg, “With all the information in front of you and the headset to free your hands, it was easy to navigate.” [Research assistant 2])*.* By preparing in advance, research assistants were able to better conduct the various research tasks, allowing them to be responsive to participants’ needs.

I tried to speak at a slow, steady pace with clarity (participant noticed and appreciated). Always asked for feedback from participants.

Research assistant 2

After asking her if my tone was appropriate, the testing went by smoothly.

Research assistant 3

They also mentioned that presession contact with the participant streamlined the implementation of the remote interaction. For instance, providing potential participants with a copy of the consent form by email was perceived by research assistants as accelerating the process and providing participants with more time to consider and provide consent that is better informed.

Research assistants also reported on their feelings of comfort while engaging in remote research. The novelty of administering the different tests and questionnaires over the phone initially generated feelings of anxiety for some. After several administrations, they reported an increase in their confidence in conducting the study, suggesting that practice improved their sense of competency (eg, “This session was directly following the other participant’s session, so I felt confident.” [Research assistant 2]). However, it was crucial to prevent excessive scheduling within a limited time frame to prevent fatigue and potential compromising of research data.

This phone call was a little more challenging for me as it was my 3rd administration and interview of the day. Therefore, I was less talkative and felt less focused.

Research assistant 3

#### Barriers to Remote Methods

Research assistants indicated that questionnaires with a wide range of response options were not well suited for remote research. Concerns about implementation included the repetitive nature of the response options and the participants’ difficulty in retaining multiple response options in their working memory.

The most difficult part was the COVID questionnaire and its response options in Likert form, because she was unsure of the answer choices, so I had to repeat them for all statements.

Research assistant 2

Research assistants also commented on several telephone-related barriers regarding practicality. These included delays in responses, overlapping communication between the participant and the researcher resulting in the decayed provision and reception of information, as well as limited settings on the telephone (eg, lack of speakerphone option). Researchers experienced technical difficulties (eg, dropped calls and diminished speech perception), which may have been a result of weak signal connection or issues with the recording software.

We spoke at the same time, and I misheard her response to the number sequence (MoCA). Fortunately, she repeated her answer.

Research assistant 1

Every 10 minutes, the line would cut off, which was disturbing.

Research assistant 1

Another important concern raised about the efficacy of the remote administration centered on language. One research assistant described apprehension about their accented speech interfering with participant comprehension because French was not the research assistant’s first language. An additional articulated barrier was communication in the case of participants living with hearing impairment, whereby research assistants were concerned that information could be misunderstood or missed. To compensate, they were careful to speak clearly at a slow, steady pace.

I had some problems with pronunciation, but I will do better next time.

Research assistant 4

Communication was difficult as participant wears hearing aid. Therefore, I had to modify my speech (slower, enhance pronunciation). I also had to repeat a lot because she could not make out my words very well.

Research assistant 3

## Discussion

### Principal Findings

The objective of this study was to examine and better understand the experiences of researchers and older participants regarding perspectives of remote research using the MoCA-Blind. As a formative study, it explored feasibility and acceptability by assessing usability, satisfaction, and potential barriers with a telephone-based cognitive screening test, with the aim of improving procedures for participants and researchers or clinicians. The findings suggested that a telephone-delivered cognitive screening tool was largely reported as feasible and acceptable for research while also identifying several barriers that ought to be addressed to enhance the experience for both participants and researchers. It is also worth noting that despite the separation of the analyses into 2 concurrent studies, the findings mapped onto the same dimensions of the framework of Bowen et al [30] (ie, barriers and facilitators); however, the content within these higher order themes retained a uniqueness, which highlighted the dyadic and experiential nature of the research interaction.

### Comparison With Prior Work

#### Facilitators to Remote Research

Perspectives of both participants and researchers converged on remote research using the MoCA-Blind as a feasible endeavor. Importantly, we found that implementing remote research was supported by a thorough evaluation of specific factors, including the technological infrastructure of researcher and participant [37], participant engagement and comfort [38], data security and privacy [38], ethical considerations [39], and the adaptability of research protocols. Careful attendance to these variables could act as a foundation to support and proliferate research on remotely delivered cognitive screening tools.

A key facilitator identified in this study was the implementation of preparatory measures by the research team, which included troubleshooting technical equipment, establishing a welcoming and cordial rapport with the participants, and ensuring transparency and clarity regarding the testing procedures. These preparatory steps played a crucial role in creating a comfortable environment for the participants, fostering a sense of trust and cooperation throughout the testing sessions. Cognitive testing is often stressful [40], and intentional attention to fostering rapport and comfort has been recommended for telephone cognitive screening [41]. The establishment of rapport with the participants likely fostered a positive atmosphere, reducing potential anxiety or discomfort associated with the research setting.

More broadly, remote methods facilitated data analysis and data interpretation. The ability to record test sessions and revisit them during the subsequent analysis phase facilitated accurate note-taking and comprehensive analysis. By preserving the exact content of the test sessions through recorded sessions, researchers were released from relying solely on their memory to score the various tasks. This possibility to revisit the recordings enhanced the quality of the data because it became possible to check its accuracy during the analysis process.

#### Barriers to Remote Research

Our participants expressed valid concerns regarding the lengthy consent process in research. To address this concern, we streamlined the consent process by either providing participants with a copy of the consent form via email prior to the testing session or by summarizing key sections of the form while ensuring that important ethical considerations (eg, risks, inconvenience, confidentiality, and privacy) were emphasized. This procedure was conceptually similar to short-form consent [42]. However, we acknowledge that these concerns may not extend to other contexts where remotely delivered cognitive tools may be used (eg, clinical practice). Participants also emphasized being mindful of session length when considering study design. This concern has been articulated previously [43], with several studies arguing that remotely delivered research can be more effortful than face-to-face contact [44-46]. Participants also noted a lack of personal connection to the process, which might lessen their interest in participation. This lack of interpersonal connection could be due to the loss of nonverbal communication [47], which may restrict rapport [48]. Interestingly, one participant suggested videoconferencing as an avenue to increase interpersonal connection; this idea has been suggested as viable in the literature [49]. Additionally, confidentiality was a noted concern for participants, in keeping with other investigations [43,50], due to risks such as the presence of others in the room [51] or participants being unsure what will happen with the data [52]. Finally, some participants mentioned opportunities for dishonesty on the part of the participant in remote research, particularly as it pertains to cognitive test performance. To our knowledge, this is the first time that participant dishonesty has been articulated as a challenge for remote research. However, this notion is consistent with the described challenges of remote academic dishonesty during the COVID-19 pandemic [53,54].

From the research assistant’s perspective, one barrier was that some instruments may not be well suited to remote modalities. This is consistent with other research recommending against the use of complicated instruments when conducting telephone interviews [55]. If the use of such tools cannot be avoided, participants could bring a pen and paper to the telephone to track response options [56] or be prompted as reminders of response categories [57]. Research assistants also discussed the potential challenges of telephone-delivered research tools with people living with hearing impairment (ie, information could be misunderstood or missed). This challenge has been described in other investigations into remote research [51,58]. It is possible that people with hearing impairment may find face-to-face methods more beneficial because they can engage in lip- and speech reading and benefit from nonverbal communication to aid in their understanding [59]. Thus, it is important to find strategies to make remote research more accessible, and remote research with videoconferencing might be ideal for persons with hearing impairment due to the possibility of lip- and speech reading and closed captioning [2]. Interestingly, our research assistants brought forth possibilities that have been conveyed in extant literature such as paying particular attention to articulation, tone, and repetition when needed [60].

Notably, participant and research assistant responses converged on 2 barriers to this remote research interaction. The first was an awareness of technical problems that are disrupting the research process. Technological difficulties continue to plague remote research [61], with problems like signal disruption [60] or lower vocal clarity [62], making our findings regarding technological disruptions perhaps unsurprising. Second, both participants and research assistants articulated concerns about remote cognitive screening administration, albeit from differing perspectives. Participants displayed some apprehension about completing the MoCA-Blind tasks correctly. Some literature on remote administration of cognitive assessments supports the presence of participant apprehension or anxiety during administration [63], while in other cases, remote methods could be seen as less anxiety-provoking [64]. Relatedly, research assistants were concerned with communication breakdowns (eg, through accented speech or overlapping verbalizations) interfering with test administration. Communication breakdowns may be more likely to occur in remote research, due to a decrease in the ability to control communication, that is, for the researcher to lead and the participant to follow [65], and may require increased attention to verbal cueing [66]. In any case, more study is needed to disentangle the complexity of how remote cognitive screening using the telephone can be accomplished most effectively.

### A Word on the MoCA-Blind

The nature of the tests conducted within the research protocol appeared to significantly influence participants’ experiences of research participation. While the simplicity and brevity of the MoCA-Blind were recognized as facilitative to the research process, it is crucial to acknowledge that this abbreviated version may not provide identical inferences compared to the full MoCA screening. By excluding vision-dependent tasks like the clock-drawing task and animal naming, important cognitive functions such as visuospatial perception, executive function, and semantic knowledge may not be fully assessed [28,29]. These tasks require visual cues and active engagement with visual stimuli, which are crucial for evaluating specific cognitive domains. Visual speech cues from the researcher are available during in-person administering that are absent over the phone. Therefore, there are vision and hearing variables at play both for the format of the test chosen and for the modality of administration. The omission of these tasks in the MoCA-Blind may limit the comprehensive evaluation of these cognitive functions. Therefore, caution should be exercised when interpreting and generalizing the results obtained using the MoCA-Blind. Researchers and clinicians should consider the specific cognitive domains being assessed and determine whether the abbreviated version aligns with their assessment goals and target population.

Additionally, study participants were recruited from a facility specializing in the health of older adults. Given that the MoCA is frequently administered in such settings to screen for mild cognitive impairment [3], it is possible that participants were already familiar with this tool. Prior exposure to the MoCA influencing their experiences through perhaps lowered anxiety or uncertainty [15,17].

### Future Directions: Diversity and Inclusivity in Research

This study has implications for the inclusion of persons with disabilities in the research process. First, by using the telephone as the delivery modality, we capitalized on a widely available device for remote communication for older adults [6]. These authors also reported that the most common problems reported in daily use of technology were visual or hearing impairments; nearly all participants owned a telephone. In addition, a study of US census data suggested that some telephone-delivered research methods do not underrepresent people with disabilities [67]. Thus, it seems likely that pursuing telephone-based research could be a way to capture the experiences of people with various types of impairments. Further, telephone-based research reduces the need for travel [43,68], promoting the inclusion of people with disabilities that may affect their independent travel [61]. With this development comes the potential for inclusion of underrepresented research populations (eg, individuals with sensory, cognitive, or mobility impairments, or a combination of these) and other marginalizing characteristics (eg, socioeconomic status and geographical location) with an aim toward representativeness and generalizability of how remote cognitive screening tools might be applied. Indeed, investigations have begun to explore the equivalency of remote MoCA administrations across various diversity dimensions including language [69] and race or ethnicity [70].

Finally, our procedure integrated several checklists (Multimedia Appendix 7) to ensure that devices, such as hearing aids, were identified and used during research sessions. We hope our materials and procedure can serve as a template for other investigators to use, extend, and adapt to facilitate the participation of people with disabilities in the research process [71]. Ultimately, telephone-based remote research has the potential to give voice to populations (eg, marginalized communities) who might otherwise go unheard [72]. This study laid the foundation for more inclusive practices emphasizing research with, and not research on, certain population. By adopting an accessible approach and actively engaging these communities in the research process, their unique perspectives, experiences, and needs can be recognized, respected, and addressed. In so doing, we can widen the base of available data on how remote cognitive screening tools might be best applied, whether in research or clinical practice.

### Limitations

This study should be considered in the context of several limitations. First, the sample size was modest, which restricts the generalizability, and may have limited achievement of data saturation. It is therefore possible that there are yet uncovered qualitative dimensions, and future studies with larger sample sizes may elucidate additional information to extend our work. However, we believe the results still have value, as some authors have cautioned against sampling thresholds for qualitative inquiry [73]. Additionally, our sample was relatively homogeneous, excluding health conditions such as cognitive or visual impairment. This sampling choice was aimed at capturing experiences in the absence of clinical complications from which to generalize future work. Such an approach permits method and procedural refinement before extending our research to more diverse and complex populations. Additionally, all participants were native French Canadian people in a health and social care system, where access to care is available without cost to the individual. Our sample was too small to speculate about gender differences in the perceived barriers and facilitators, which should be explored in future studies. Furthermore, it is worth mentioning that only 1 individual scored below the cutoff score for the MoCA, indicating a limited range of cognitive variability among our participants. Our study did not gather data on the participants’ educational and socioeconomic backgrounds, which could have provided valuable insights into their profiles to contextualize the findings and uncovered different barriers and facilitators to remote research. We note that the lack of information on participant characteristics limited the generalizability of the results.

Another limitation was the absence of comparative data between telephone and in-person administration. By incorporating data from both remote and in-person research contexts, we would have been able to examine potential differences, advantages, and challenges associated with each approach. Although this research explored the overall experience of remote research, it was restricted to the use of telephone only versus the use of videoconferencing for remote research. However, it is possible that the insight from the participants may vary depending on the nature of the study and the individual study materials and tasks. Factors such as the complexity and length of study protocol and materials, the nature of the tasks, and the level of participant engagement may all contribute to potential variations in participants’ feedback and perceptions. Finally, the MoCA-Blind was used as a vehicle to understand the experience of telephone screening research. This measure was originally conceptualized for individuals living with visual impairment; however, none of the participants in our study reported visual impairment. Consequently, the absence of individuals with visual impairments in the sample limits our ability to fully capture the specific challenges and experiences they may encounter in remote research scenarios. This measure is, however, suitable for telephone administration, given that it does not require vision and only depends on hearing ability.

Further, while qualitative analysis of field notes from the research assistants provided insights into their perspective, the form of our open-ended questions may pose a limitation. The notes may lack depth and did not generally allow for follow-up questioning, posing a methodological bound to our understanding of their experiences and perspectives.

Finally, this study provided insights into the MoCA-Blind’s utility as a cognitive screening tool for research purposes. However, we were unable to draw conclusions with respect to the feasibility of the tool as a clinical instrument. Indeed, during the COVID-19 pandemic, uncertainty was suggested around the tool with respect to its remote clinical use [19]. In addressing this limitation, future work could extend our findings into a clinical interaction (eg, seeking patient perspectives following a clinical encounter) or could incorporate clinician perspectives in place of research assistants.

### Conclusions

This study investigated the experiences of administering and receiving a remotely delivered telephone-based cognitive screening tool in the research setting. Barriers and facilitators to the research process emerged from both participants and researchers. Participants identified short instrument length, convenience, and presession contact as facilitative; barriers included the length of the interaction, some tasks being more challenging on the phone, and the potential for participant dishonesty. On the other side of the interaction, research assistants noted the process was facilitated by instrument length, rapport building, session preparation or recording, and comfort with the protocol; barriers included items with too many response options, telephone issues (eg, response delays), and concerns about participant comprehension. Despite some limitations (eg, sample and the optimal conditions, such as participants without sensory or cognitive limitations), the findings provide support for the MoCA-Blind as a feasible and acceptable tool for research inquiry, with barriers that can be conceptualized as areas for future development.

## Appendix

Multimedia Appendix 1 Telephone use questionnaire.

Multimedia Appendix 2 COVID-19 questionnaire.

Multimedia Appendix 3 Qualitative interview guide.

Multimedia Appendix 4 Qualitative assessment form for researchers.

Multimedia Appendix 5 Demographic questionnaire for research assistants.

Multimedia Appendix 6 COREQ (Consolidated Criteria for Reporting Qualitative Research) checklist.

Multimedia Appendix 7 Experiment checklists.

## Abbreviations

COREQ: Consolidated Criteria for Reporting Qualitative Research
MoCA: Montreal Cognitive Assessment
