# Supplementary material for: Exploring the Qualitative Experiences of Administering and Participating in Remote Research via Telephone Using the Montreal Cognitive Assessment-Blind: Cross-Sectional Study of Older Adults
Source: JMIR Form Res. 2024 Nov 15;8:e58537. doi: 10.2196/58537 (PMC11607555; doi:10.2196/58537)
Supplement: Multimedia Appendix 1 [file formative_v8i1e58537_app1.docx]

1. What type of phone do you typically use to make phone calls?
   1. Corded landline/home phone
   2. Mobile phone
   3. Other, please specify. ______________
2. When answering the phone, do you:
   1. Hold it to your ear
   2. Hands-free/speakerphone
   3. With earphones/headphones
   4. Other, please specify. ________________
3. (If mobile phone) What is your phone carrier?
   1. Bell
   2. Fido
   3. Rogers
   4. Virgin
   5. Telus
   6. Videotron
   7. Other, please specify. _____________
   8. Do not know
4. How long have you been using this device?
   1. Less than 6 months
   2. Between 6 months and a year
   3. Between 1 and 3 years
   4. Do not know/No answer
5. How frequently do you use your phone to make calls?
   1. Daily
   2. Almost every day
   3. Once a week
   4. Never
   5. Do not know/Prefer not to say
6. Do you often require help in order to use efficiently your landline/mobile phone?
   1. Yes
   2. No
   3. Prefer not to say
7. Do you enjoy making phone calls?
   1. Yes
   2. No
   3. Indifferent
   4. No answer
8. Do you often encounter technological issues when you are using your device?
   1. Yes
   2. Sometimes
   3. No
   4. Don’t know/No answer
9. How important is technology in your life?
   1. Very important
   2. Important
   3. A little important
   4. Not important
   5. Don’t know/No answer
