# Supplementary material for: Exploring the Qualitative Experiences of Administering and Participating in Remote Research via Telephone Using the Montreal Cognitive Assessment-Blind: Cross-Sectional Study of Older Adults
Source: JMIR Form Res. 2024 Nov 15;8:e58537. doi: 10.2196/58537 (PMC11607555; doi:10.2196/58537)
Supplement: Multimedia Appendix 2 [file formative_v8i1e58537_app2.docx]

1. **Have you been hospitalized?**

1 – NO

2 – YES

8 – Don’t know/No answer

9 – Prefer not to answer

1. **In the past month have you had testing to determine if you have COVID-19?**

1 – No

2 – YES

8 – Don’t know/No answer

9 – Prefer not to answer

1. **Was the test positive?**

1 – NO

2 – YES

3 – Results not available yet

8 – Don’t know/No answer

9 – Prefer not to answer

1. **Over the last 2 weeks, how often have you been bothered by the following problems?**

|  | Not at all | Several days | More than half the days | Nearly everyday | Don’t know/No answer | Prefer not to answer |
| --- | --- | --- | --- | --- | --- | --- |
|  | 0 | 1 | 2 | 3 |  |  |
| Feeling nervous, anxious or on edge |  |  |  |  |  |  |
| Not being able to stop or control worrying |  |  |  |  |  |  |
| Worrying too much about different things |  |  |  |  |  |  |
| Trouble relaxing |  |  |  |  |  |  |
| Being so restless that it’s hard to sit still |  |  |  |  |  |  |
| Becoming easily annoyed or irritable |  |  |  |  |  |  |
| Feeling afraid as if something awful might happen |  |  |  |  |  |  |
