# Supplementary material for: Exploring the Qualitative Experiences of Administering and Participating in Remote Research via Telephone Using the Montreal Cognitive Assessment-Blind: Cross-Sectional Study of Older Adults
Source: JMIR Form Res. 2024 Nov 15;8:e58537. doi: 10.2196/58537 (PMC11607555; doi:10.2196/58537)
Supplement: Multimedia Appendix 3 [file formative_v8i1e58537_app3.docx]

**Qualitative interview guide for participants**

**Study title:** Test-retest variability of cognitive assessments: Comparing in-person with telephone assessments during remote-research

*We would now like to ask you a few questions about your experience of participating in a research study over the phone. We simply want to understand your experience, how you felt during this phone call, what you liked and what you did not like about this procedure. Please do not take into consideration how you think you performed on the tests but simply reflect on your overall experience.*

**Question 1:** Can you describe how it felt to be tested over the phone?

Probes: anticipation, motivation to participate, psychological state, physical state

*Goal: learn about their experience of being tested over the phone.*

**Question 2:** What is your opinion on the use of telephone for researchers to conduct testing?

Probes: versus home visit/practicality, time, task explanation/comprehension, social aspect, environmental factors/distractors/concentration, observation pressure

*Goal: learn about their subjective perspective of remote research.*

**Question 3:** How would remote testing influence your future interest in participating in research experiments? Why?

Probes: increased/decreased interest, cessation, first or second choice or indifferent; etc.

*Goal: learn if remote research would be a deterrent in future participation in research.*

**Question 4:** What would be your suggestions to us researchers so that we adapt this testing session to enhance your experience of testing over the phone?

*Goal: acquire tips from participants to improve future remote research.*

**Question 5**: We sent you instructions prior to our testing session over the phone. Can you tell me how these instructions were helpful or not?

Probes: what they were (recall); helped in preparation; were followed by participant; were clear; telephone, researcher guidance, organization, we should have mentioned, organization of testing session.

*Goal: learn if our efforts were successful in enhancing the remote research participation.*

**Question 6:** What other factors or aspects of remote testing do you believe we would need to consider for our future participants?

*Goal: learn about the various aspects to consider for future remote research.*

**Last Question:** Thank you for all that valuable information, is there anything else you’d like to add before we end?
