# Supplementary material for: Exploring the Qualitative Experiences of Administering and Participating in Remote Research via Telephone Using the Montreal Cognitive Assessment-Blind: Cross-Sectional Study of Older Adults
Source: JMIR Form Res. 2024 Nov 15;8:e58537. doi: 10.2196/58537 (PMC11607555; doi:10.2196/58537)
Supplement: Multimedia Appendix 4 [file formative_v8i1e58537_app4.docx]

**Interview qualitative assessment – test administrators**

1. **Field notes:** Please provide a general description of the testing session (i.e., salient/unusual elements, the participant’s attitude, the course of the call, etc.)

____________________________________________________________________________________________________________________________________________________________

______________________________________________________________________________

______________________________________________________________________________

______________________________________________________________________________

1. **Experimenters’ notes:** Please write your comments and suggestions on the following aspects based on your phone call (consider both your experience and your perception of the experience of the participants). Please note that the following points only serve as prompts: if you have already addressed them in section A, please skip:

**Preparation:**_________________________________________________________________________________________________________________________________________________ ______________________________________________________________________________

**Technology:**_________________________________________________________________________________________________________________________________________________ ______________________________________________________________________________

**Time:**____________________________________________________________________________________________________________________________________________________________________________________________________________________________________

**Testing environment: ____________**________________________________________________________________________________________________________________________________________________ ______________________________________________________________________________

**Interruptions/distractions:**___________________________________________________________________________________________________________________________________________________________________________________________________________________

**Troubleshooting/guidance:**__________________________________________________________________________________________________________________________________________________________________________________________________________________

**Test/interview administration:** ____________________________________________________________________________________________________________________________________________________________________________________________________________________________

**Comfort** (prompts: physically, emotionally, workload burden, multitasking, trouble shooting, helplessness):______________________________________________________________________________________________________________________________________________________________________________________________________________________________

**Efficiency:** __________________________________________________________________________________________________________________________________________________________________________________________________________________________________________

**Your impression of the participant (i.e.., attitude):** __________________________________________________________________________________________________________________________________________________________________________________________________________________________________________

**Other, please specify:** __________________________________________________________________________________________________________________________________________________________________________________________________________________________________________
