# Supplementary material for: Exploring the Qualitative Experiences of Administering and Participating in Remote Research via Telephone Using the Montreal Cognitive Assessment-Blind: Cross-Sectional Study of Older Adults
Source: JMIR Form Res. 2024 Nov 15;8:e58537. doi: 10.2196/58537 (PMC11607555; doi:10.2196/58537)
Supplement: Multimedia Appendix 5 [file formative_v8i1e58537_app5.docx]

1. ID number
2. First name: Last name:
3. Phone number
4. Date of birth: DD_MM_YYYY
5. Age
6. Sex at birth
7. Gender
8. Language 1
9. Language 2
10. Status (i.e., student, professional)
11. Education achieved
