# Supplementary material for: Exploring the Qualitative Experiences of Administering and Participating in Remote Research via Telephone Using the Montreal Cognitive Assessment-Blind: Cross-Sectional Study of Older Adults
Source: JMIR Form Res. 2024 Nov 15;8:e58537. doi: 10.2196/58537 (PMC11607555; doi:10.2196/58537)
Supplement: Multimedia Appendix 7 [file formative_v8i1e58537_app7.docx]

1. **Checklist #1 (during first phone call):**
   1. Are you wearing a hearing aid? 1 – YES 2 – NO
   2. When making phone calls, do you wear your hearing aid? 1- YES 2- NO 3 – SOMETIMES
   3. If so, in what ear? 1 – RIGHT 2 – LEFT 3 – BOTH
   4. Please make sure that your hearing aid battery is full before our call appointment.
   5. Please make sure that your phone battery is full before our call appointment.
   6. Please ensure that you will be alone in a room, without any distractions (tv, other people, etc.) **for 1 hour.**
   7. Please make sure you have the equipment necessary to use your preferred method of phone use during our call (headphones).
   8. Please have a pen handy, whatever kind of pen.
2. **Checklist #2 (during second phone call):**
   1. Are you wearing your hearing aid (if this applies)?

- *If no, ask if they would more comfortable wearing it during the phone call.*
  1. Is your hearing aid battery charged?
  2. Are you in an environment where you will not be interrupted for at least an hour?
  3. Is your device’s battery charged?
  4. Can you please confirm how you will be using the phone (hands-free, speaker, headphones)?
  5. Do you have a pen?

1. **Verification questions (asked twice during second phone call):**
   1. Are you still hearing me clearly? 1. YES 2- NO
   2. Have you switched phone call mode (speaker, hands-free, headphones)? 1- YES 2-NO
   3. Please let me know if you need a break at any time.
